# Cultural Diversity and Saccade Similarities: Culture Does Not Explain Saccade Latency Differences between Chinese and Caucasian Participants

**DOI:** 10.1371/journal.pone.0094424

**Published:** 2014-04-07

**Authors:** Paul C. Knox, Felicity D. A. Wolohan

**Affiliations:** Eye and Vision Science, Institute of Ageing and Chronic Disease, University of Liverpool, Liverpool, United Kingdom; Macquarie University, Australia

## Abstract

A central claim of cultural neuroscience is that the culture to which an individual belongs plays a key role in shaping basic cognitive processes and behaviours, including eye movement behaviour. We previously reported a robust difference in saccade behaviour between Chinese and Caucasian participants; Chinese participants are much more likely to execute low latency express saccades, in circumstances in which these are normally discouraged. To assess the extent to which this is the product of culture we compared a group of 70 Chinese overseas students (whose primary cultural exposure was that of mainland China), a group of 45 participants whose parents were Chinese but who themselves were brought up in the UK (whose primary cultural exposure was western European) and a group of 70 Caucasian participants. Results from the Schwartz Value Survey confirmed that the UK-Chinese group were culturally similar to the Caucasian group. However, their patterns of saccade latency were identical to the mainland Chinese group, and different to the Caucasian group. We conclude that at least for the relatively simple reflexive saccade behaviour we have investigated, culture cannot explain the observed differences in behaviour.

## Introduction

Cognitive and behavioural differences between human populations have been reported in a wide range of studies [1]–[6], contributing to the emergence of “cultural neuroscience” [7]–[10]. In a number of these studies, eye movement metrics have been used to investigate differences between participant groups [11]–[15].

We recently demonstrated a difference between Chinese and Caucasian (white British) participants in a reflexive saccade task [16]. When a blank period (“gap”) is introduced between central fixation target offset and eccentric saccade target presentation, saccade latency is reduced (the “gap effect”) in both humans [17] and in non-human primates [18]. Gaps also encourage the production of a particular class of low-latency, visually-guided “express” saccade (ES). ES formed a distinct peak in saccade latency distributions in monkeys [18], [19], but only occasionally did so in humans, leading to considerable controversy [20]–[23]. However, in the monkey, it was shown that they were critically dependant on the integrity of the superior colliculus [19] and occurred when a general reduction in inhibition allowed the visual (target onset) response burst in collicular saccade-related neurons to trigger saccades directly [24], [25]. This contrasts with the more usual pattern in which the visual burst is followed (later in time) by a second motor burst which triggers the saccade. These findings provided evidence that ES are a neurophysiologically distinct type of saccade. While ES production is encouraged by gaps, it is inhibited by leaving the central fixation target illuminated when the eccentric saccade target appears (the “overlap” paradigm). However, using overlap tasks, we found that a much higher proportion of Chinese participants persisted in producing high numbers of ES compared to a Caucasian group; that is, in the Chinese group there was a much higher proportion of “express saccade makers” (ESMs: healthy, naïve participants, who in overlap tasks produce >30% express saccades [26], [27]). We subsequently confirmed this unexpectedly high proportion of ESMs in a second, larger group of Chinese participants, and demonstrated that the Chinese ESMs were also compromised on a voluntary, antisaccade task [28]. In conditions that required the inhibition of reflexive prosaccades (error saccades towards a suddenly appearing target in this context), they were less able to inhibit error prosaccades than would be expected (they had a significantly increased directional error rate) and many of those error saccades had much lower latencies than error saccades in non-ESM participants.

The Chinese participants tested in our earlier experiments were both ethnically and culturally Chinese; they were all recruited and tested in China. It was thus impossible to separate the issue of culture from the issue of ethnicity (or nationality), and we had no independent measure of “culture”. This is also true of a number of other studies in which culture has been claimed to be modifying some process or behaviour [11], [12], [14], [15]. So in both our and these other studies, it is premature to claim that differences in culture explain the functional differences observed. One means of addressing this issue is to recruit participant groups of identical ethnic background, but whose primary cultural exposure differs. Experiments of this type have in fact tended to suggest that culture has a relatively weak influence on at least some aspects of behaviour [29], [30]. We therefore repeated our original experiment in two Chinese groups: one composed of participants born and educated in China, the second composed of participants born in the UK to Chinese parents (or who moved to the UK at a young age) and who were educated in the UK.

Rather than simply assume that two groups recruited as described would be culturally different, we wished to try and establish this by finding some means of assessing or “measuring” the culture of the groups. This is not a simple matter, particularly given the difficulty of defining what culture is [31], [32] and other recent criticisms of cultural neuroscience [9], [33]. A reasonable definition of culture would include references to shared knowledge, values, beliefs, practices and perhaps even artefacts, of different human groups. The groups need not necessarily be defined in national or geographical terms, although participant groups have often been defined in these terms, and assumptions then made about culture or cultural differences[32], [33]. However, in any given group measuring a long list of features (even where suitable measurement instruments are available), in order to “measure” culture, is problematic.

An alternative approach is represented by “values theory” [10], [34], [35]. Schwartz values theory derives a limited number of values that are claimed to be present in all human cultures because they are grounded in the needs of individuals as biological organisms, the requirements imposed by the need for coordinated social interaction, and the needs of the survival and welfare of groups. To identify these values, and determine their relative importance within different groups, the Schwartz Value Survey (SVS) was developed and has been widely used [36]–[38], including in a number of cultural neuroscience studies [39], [40].

We therefore collected SVS and oculomotor data from two groups of Chinese participants and also a Caucasian comparison group. If culture is the key driver of the oculomotor differences we have observed, then assuming the British-born/educated Chinese group is in fact culturally distinct from the Chinese-born Chinese group, their oculomotor performance should also be different, and more closely resemble that of the Caucasian group.

## Methods

### Ethics statement

These experiments were approved by the University of Liverpool Research Ethics Committee. All participants provided their written, informed consent and experiments were performed in accordance with the ethical standards laid down in the Declaration of Helsinki (as modified 2004).

### Participants

Three groups of healthy, naïve, adult participants with normal or corrected to normal visual acuity were recruited in and around the University of Liverpool, and tested in Liverpool. The first group consisted of 70 participants born and educated in China, studying in Liverpool as overseas students (median age: 22y; 15 male; Group CC). The second group consisted of 45 participants whose parents were Chinese, but who themselves were born in the UK (or moved to the UK early in life), and who were educated in the UK (median age: 22y; 19 male; Group BC). The third group consisted of 70 Caucasian (ie white British) participants. Note that this group included 38 participants whose data were reported previously [16], supplemented by a further 32 participants recruited and tested subsequently (median age of group of 70: 23y; 27 male; Group WB). Participants were paid £5/hr for their time.

**Figure 1:**
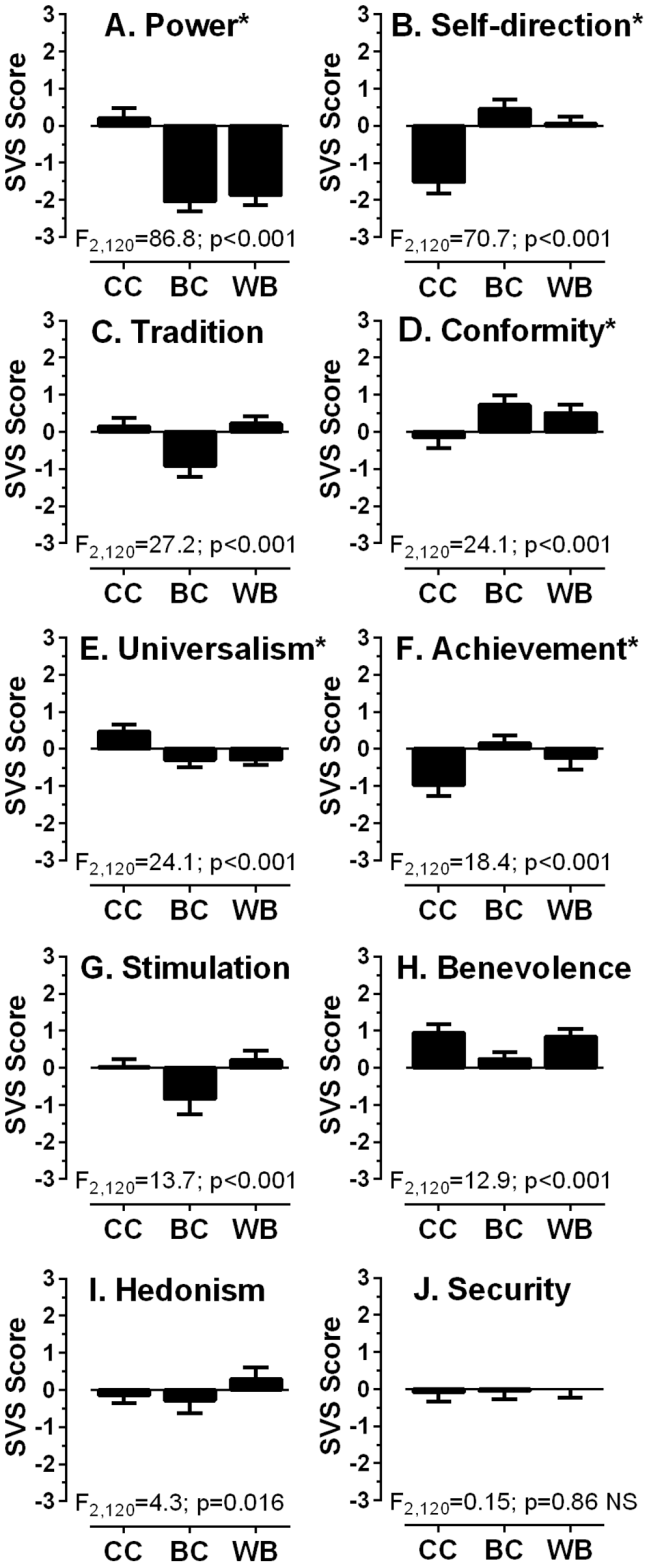
Plots of Schwartz Value Survey (SVS) results. Mean±95% CI value scores for each of the ten “values” for each participant group are plotted (CC: participants born and educated in China; N = 38; BC: Chinese ethnic background, born in the UK [or moved in early life], educated in the UK, N = 43; WB: Caucasian comparison group, N = 42). Group scores were compared with a one-way ANOVA for each value. Statistically significant results were observed for all values except “Security”. Marked values (*) are those for which post-hoc testing demonstrated that the BC and WB groups were similar and statistically distinct from the CC group. This was true for 5/9 values returning statistically significant ANOVA results.

After completing a short demographic questionaire, 43 BC and 42 WB participants also completed the Schwartz Value Survey (SVS[37]; see also Table S1 and Figure S1 in File S1) in English, while 38 of the CC particpants completed the Mandarin version of the same questionaire, as reccomended in the SVS Users Manual[41]. The SVS is a 57-item questionairre from which ten value scores (see Figure 1) are generated by approriate grouping of items (Table S1 in File S1). Individuals rate each item for its importance as a guiding principle in their life on a Likert scale ranging from -1 (against my values) to 7 (of supreme importance). The data were cleaned and individual scores computed in accordance with instructions in the Users Manual. The mean of each participant's ratings across all items was calculated to provide the mean rating score; this was then subtracted from each individual item rating for that participant, correcting for individual response bias. The ten value scores were then computed and group mean scores for each value were generated for each of the participant groups.

### Apparatus and stimuli

Horizontal eye movements were recorded binocularly with the same miniaturised head-mounted infrared saccadometer (Advanced Clinical Instrumentation, Cambridge, UK) used in previous experiments [16], [28]. This samples infrared reflectance signals at 1 KHz, and low-pass filters them at 250 Hz with 12-bit resolution. The device incorporates three low-power red lasers projecting red 13 cd/m^2^ target spots subtending approximately 0.1°, in a horizontal line, centrally and at 10° to left and right of centre. As the stimuli move with the head, participants were not head-fixed; they sat in a comfortable position approximately 1.5 m in front of a near-white surface.

Procedures were identical to those used previously [16]; participants completed 2×200 gap and 2×200 overlap trials. In gap trials, after a randomised fixation period of 1 s–2 s, the central fixation target was extinguished 200 ms prior to the appearance of the saccade target, presented randomly 10° either to the right or left. In overlap tasks, the central fixation target remained illuminated throughout the trial. Again, after the randomised fixation period the saccade target appeared. Regardless of trial type, participants were instructed to saccade to the eccentric target as soon as it appeared, pause, blink and then return their gaze position to the centre in preparation for the next trial. The order of the four blocks was counterbalanced across participants.

### Oculomotor Analysis

Data were stored on the Saccadometer handset, and downloaded for offline analysis using the supplied software (Latency Meter 4.0). The latency and amplitude of each saccade was collated and saccade latency distributions were calculated for each individual participant. We excluded from the analysis saccades with a latency of less than 50 ms or more than 500 ms. Median latency and mean saccade amplitude were calculated for each participant. We also calculated the percentage of express saccades (saccades with latency in the range 80 ms to 130 ms). We defined participants who had greater than 30% of their saccades with latency in this range in overlap conditions as “express saccade makers” (ESMs) [14]–[16].

## Results

### Comparison of groups – group characteristics and SVS results

Within group BC, 31% reported no or poor spoken Mandarin or Cantonese, 20% reported it as fair and 47% good or fluent; these participants reported much poorer written Mandarin or Cantonese (64% none/poor; 16% fair; 17% good or fluent). SVS analysis (Figure 1) suggested that across a number of SVS values the BC and WB groups tended to be similar, and different to the CC group. Using an omnibus repeated-measures ANOVA with “value score” as a within and “group” as a between subjects factor, we found that value score generated a statistically significant result (F_9,1080_ = 51.2; p<0.001), while group did not (F_2,120_ = 2.4; p = 0.09); however, there was a statistically significant value x group interaction (F_18,1080_ = 21.4; p<0.001). Given this, we compared the group mean scores for each of the ten SVS values using one-way ANOVAs (see Figure 1). All values except “Security” returned statistically significant results. For each of the remaining nine values, a post-hoc test (Tukey HSD) was used to examine the differences between groups. For “Power”, “Self-direction”, “Conformity”, “Universalism” and “Achievement” the BC and WB scores were not statistically different (p>0.05) while there were statistically significant differences between both BC and CC, and WB and CC scores (p = 0.001 or less). There was only one value (“Benevolence”) in which the BC and WB groups were statistically significantly different (p<0.001) with no difference between the BC and CC groups (p>0.05). Thus the overall pattern of value scores suggested that the BC and WB groups were broadly similar, and both were different to the CC group.

### Comparison of groups – oculomotor analysis

The CC group yielded a total of 25,349 analysable gap and 25,355 overlap trials (91±9% and 91±7% of participants' gap and overlap trials, respectively), the WB group 25,473 analysable gap and 25,273 overlap trials (91±10% and 90±12%) and the BC group 16,548 analysable gap and 16,978 overlap trials (92±11% and 94±8%).

Saccade latency, amplitude and the proportion of express saccades (%ES) was calculated for each of the three participant groups (Table 1). For latency and %ES, the two Chinese groups were similar, for both gap and overlap conditions, and appeared to be different to the Caucasian group. Amplitude was similar between groups and across conditions. For each of these parameters we ran repeated-measures ANOVAs, treating condition (gap vs overlap) as a within-subjects factor, and group (CC vs BC vs WB) as a between subjects factor. For latency, both condition (F_1,182_ = 1002.1; p<0.001) and group (F_2,182_ = 14.7; p<0.001) exhibited statistically significant differences. Post-hoc testing demonstrated that the two Chinese groups were similar, with the difference driven by the Caucasian latency being longer in both gap and overlap conditions. We observed a similar pattern for %ES (condition: F_1,182_ = 771.4; p<0.001; group: F_2,182_ = 11.4; p<0.001), with the two Chinese groups exhibiting similar proportions of ES, and the difference being driven by the lower proportion in the Caucasian group. For amplitude, there was no statistically significant difference in either factor (both F<1, p>0.4). In addition we calculated the magnitude of the gap effect for each participant and compared this across groups with a one-way ANOVA. This was statistically significant (F_2,182_ = 12.4; p = 0.002), with this again being driven by the difference between the Caucasian and the two Chinese groups (CC v BC: nsd; WB v CC p<0.001; WB v BC p = 0.002).

**Table 1:** Summary saccade parameters for the three participant groups.

Group	Gap			Overlap			
	Latency (ms)	Amplitude (deg)	%ES	Latency (ms)	Amplitude (deg)	%ES	Gap Effect (ms)
CC	120±16	11.0±2.3	50±21	165±25	10.8±2.2	20±16	45±17
BC	117±14	10.6±1.2	52±18	165±26	10.5±1.7	18±16	47±19
WB	127±19	10.7±1.9	42±23	189±31	10.7±1.9	13±12	61±27

CC: Participants born and educated in China, studying in the UK (N = 70); BC: Chinese ethnic background, born in the UK (or moved as infants), educated in the UK (N = 45); WB: Caucasian (white British) comparison group (N = 70). Intersubject mean median (±SD) saccade latency, intersubject mean amplitude, and mean percentage of express saccades (%ES) is shown for gap and overlap conditions. The final column shows the IS mean gap effect (Overlap-Gap latency).

**Figure 2:**
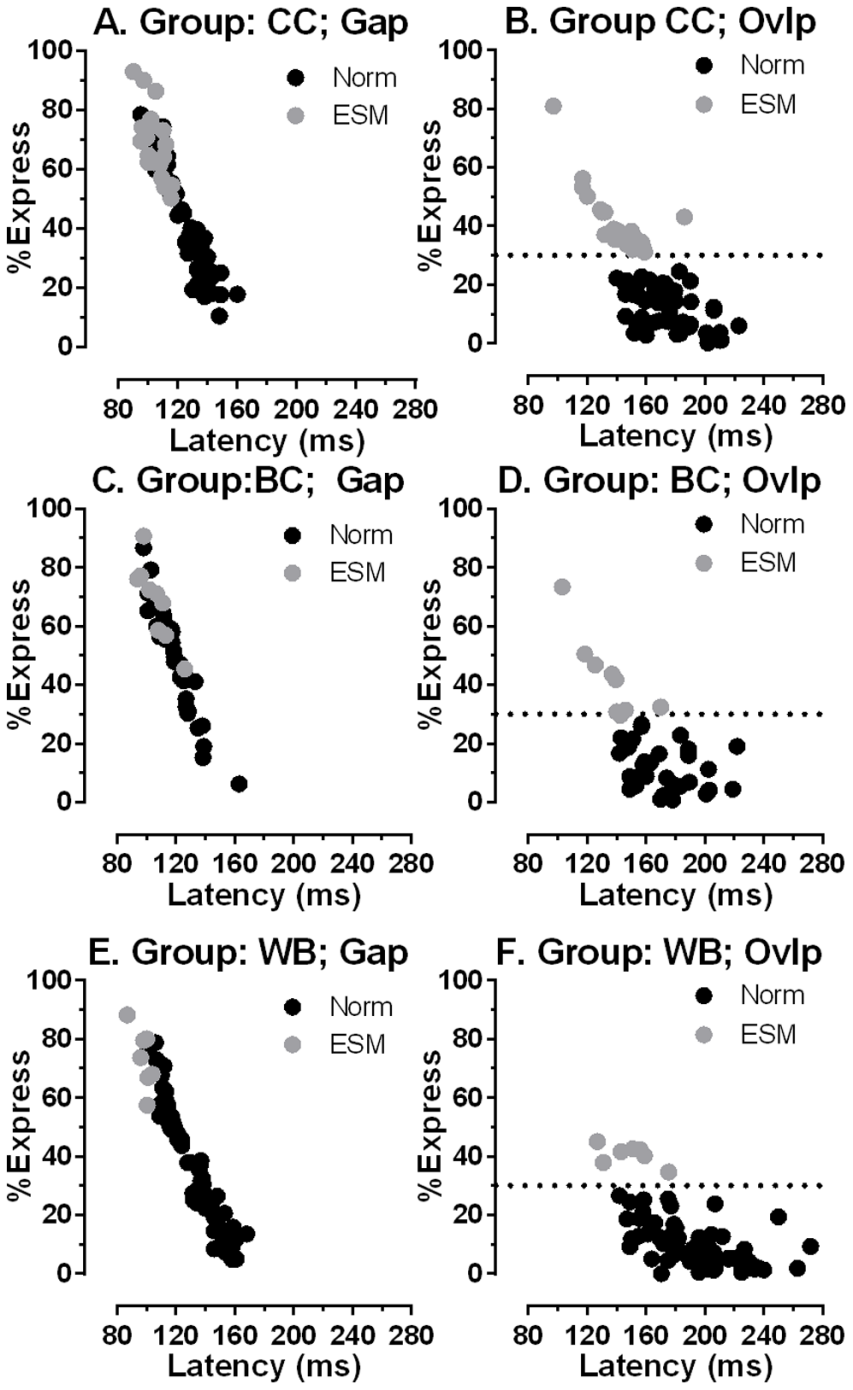
Express saccades and median saccade latency. The percentage of express saccades (%Express) is plotted against median saccade latency in gap (A,C,E) and overlap (B,D,F) conditions for the three participant groups (CC: A,B; BC: C,D; WB: E,F). • ESMs (participants with >30% express saccades in overlap conditions); • Norm (non-ESM participants). The horizontal line in overlap plots (b,d,f) illustrates the 30% criterion above which participants were defined as ESMs.

Plots of the percentage of ES against median latency in gap and overlap conditions for the three groups are shown in Figure 2. In gap conditions the groups were broadly similar. In overlap conditions the longer median latencies in the WB group and generally lower percentage of ES can be observed. A number of the WB group met the criterion defining an ESM. However, the proportion of ES in the Chinese groups was higher than that observed in the WB group.

**Figure 3:**
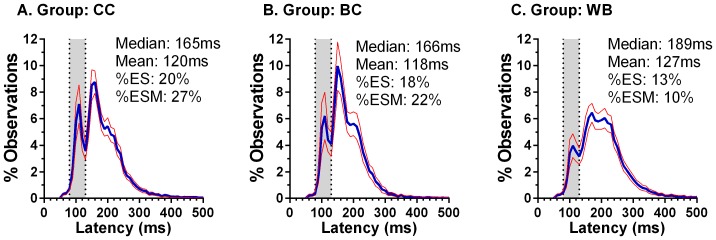
Average (mean±95%CI) percentage latency distribution histograms for the three participant groups for overlap data. Bin width = 10 ms. Mean and median latency for these distributions are also shown, along with the percentage of observations falling in the ES range (%ES; range 80–130 ms, shown by vertical dotted lines), and the proportion of ESM participants in that group (%ESM).

Given that, as we observed previously [16], it was in overlap conditions that the clearest differences emerged between the two Chinese groups and the Caucasian group, we examined the distribution of saccade latency in these conditions by calculating average percentage distributions for each group. Each bin value is the mean for that bin across all participants in that group (with 95% confidence limits; Figure 3). In both Chinese groups (Figure 3 a. CC; b. BC) there were two clear early peaks (centred at 110 ms and 160 ms) with a third, less discernible, peak at 200 ms. In the Caucasian distribution (Figure 3c. WB) there were noticeably smaller peaks at 110 ms and 170 ms. For each group distribution we took the peak and two neighbouring bin values for the ES peak (peak at 110 ms in all three groups), the fast regular peak [42](CC:160 ms; BC 150 ms; WB 170 ms) and the slow regular peak (CC and BC: 200 ms; WB 210 ms). We then performed a repeated measures ANOVA treating peak (ES v FR v SR) and bin (1,2,3) as within subjects and group (CC v BC v WB) as between subjects factors. Peak (F_2,181_ = 27.6, p<0.001) and bin (F_2,181_ = 26.5, p<0.001) generated significant results as did the peak x group (F_4,364_ = 4.4, p = 0.002) and bin x group (F_4,364_ = 12.7, p<0.001) interactions. Importantly group was also statistically significant (F_2,181_ = 23.1, p<0.001). Post hoc tests for group demonstrated statistically significant differences between the WB and both Chinese groups (p<0.001 in both cases), with no statistically significant difference between the CC and BC groups (p = 0.147).

### Proportions and characteristics of ESMs

We investigated the extent to which the group differences described above were driven by the underlying proportions of ESMs in each group. ESMs were indeed more common in the two Chinese groups (CC: 19/70, 27%; BC 10/45, 22%) than in the Caucasian group (WB: 7/70, 10%). An overall comparison of the proportions of ESMs returned a statistically significant result (Χ^2^ = 6.85; p = 0.033). While the proportion of ESMs was not statistically different between CC and BC groups (Χ^2^ = 0.352, p = 0.55), there was a difference between CC and WB groups (Χ^2^ = 6.8, p = 0.009). The difference in proportions in BC and WB groups fell short of statistical significance (Χ^2^ = 3.25, p = 0.072).

**Figure 4:**
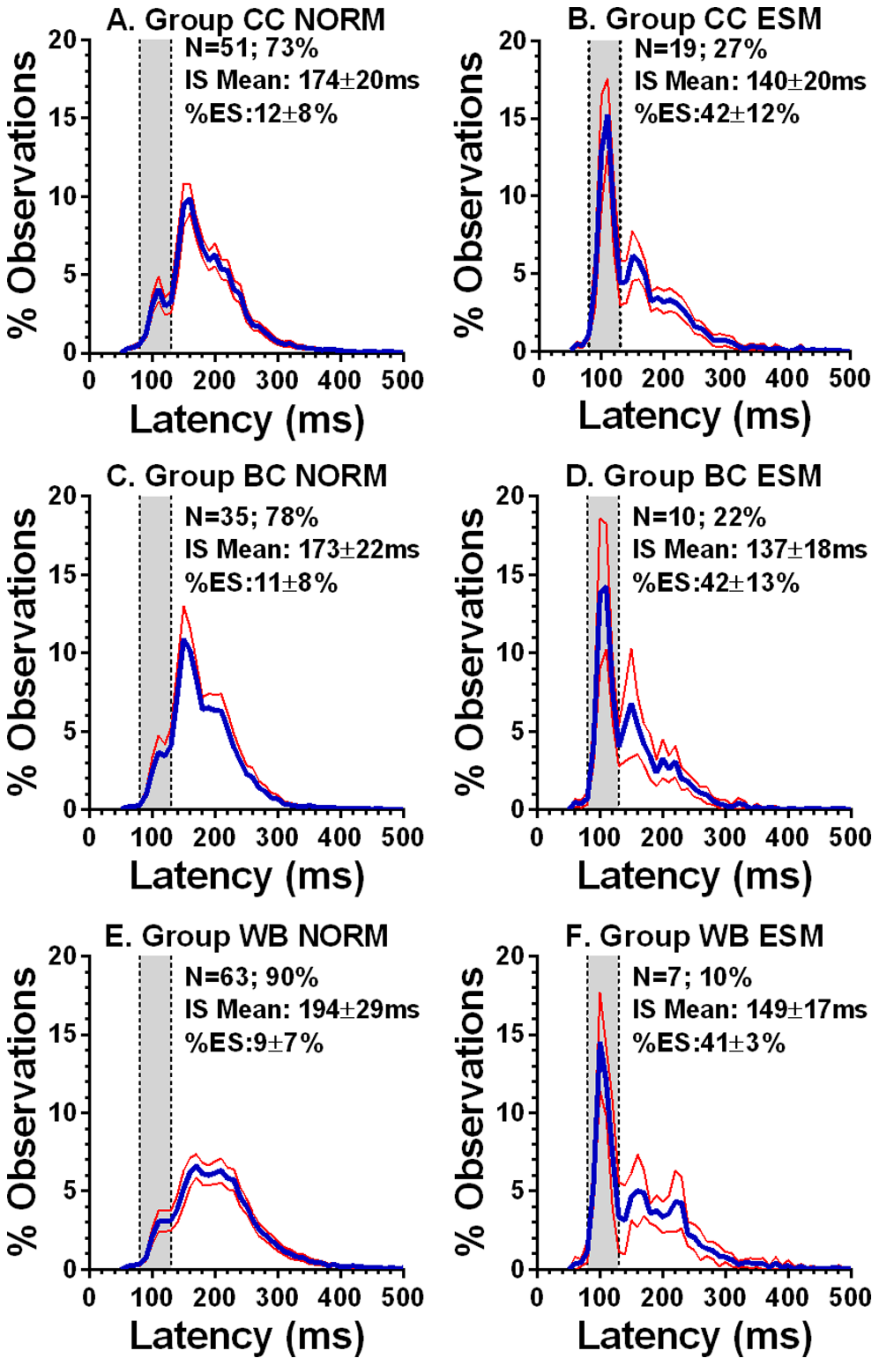
Overlap average latency distributions for ESMs and “normal” participants. Average (mean±95%CI) percentage latency distributions in overlap conditions for each group divided into Normal participants (Norm; A,C,E) and ESMs (B,D,F). Each panel also shows the number and percentage of each participant type within that group, the intersubject mean median latency (±SD), and the mean (±SD) percentage of express saccades.

Each of the three participant groups was divided into ESMs (>30% of saccades in overlap conditions with latency in the range 80–130 ms) and “normals”, and average distributions recalculated (Figure 4). The Caucasian ESM distribution (Figure 4f) was very similar to those from the two Chinese groups (CC: Figure 4b; BC: Figure 4d). These confirmed that the ESMs in the Chinese groups, while more numerous, performed in a very similar manner to the small group of Caucasian ESMs we were able to test in this experiment.

## Discussion

Differences in perception [5], [6], cognition [29], [40] and behaviour [12], [14], [15] are consistently observed between participant groups drawn from different human populations. We have confirmed in the present experiment that within Chinese participant groups, a much higher proportion of participants than expected persisted in executing large numbers of express saccades (visually guided saccades with latency in the 80 ms–130 ms range) in circumstances in which these are normally discouraged (overlap tasks in which the central fixation target is present when the saccade target appears). Using the criterion employed previously, we defined those participants as “express saccade makers” (ESMs). In the two Chinese groups we confirmed a high proportion of ESMs (CC:27%; BC:22%) compared to the Caucasian group (10%). These proportions are similar to those we reported previously for Chinese participant groups recruited and tested in mainland China using the same tasks and equipment (29%[16] and 22%[28]).

Statistically significant differences were observed in average (mean) median latency (but not amplitude) between the Chinese and Caucasian groups across conditions (Table 1). The difference was small in gap conditions (CC vs WB:7 ms; BC vs WB:10 ms), but larger in overlap conditions (CC and BC vs WB:24 ms) because of the lower latencies in the Chinese groups. This meant that the gap effect was consistently smaller in both Chinese groups compared to the Caucasian group. The reason for the lower median latencies in the Chinese groups was a significantly higher proportion of express saccades and fast regular saccades, as evidenced in the average distributions for overlap conditions (Table 1, Figure 3). The similarity of the distributions (as well as median latency) for the two Chinese groups, and the difference between these and the Caucasian group, is clear. Note also the consistency between the current and our previous results for Chinese participants recruited and tested in China (eg compare Figure 1c & d in [28] with Figure 4a-d). Both the occurrence and the timing of the express, fast and slow regular peaks in saccade latency distributions for the type of saccade task used in these experiments is consistent with what has been reported previously[42], [43].

The usefulness of classifying some participants as ESMs using the 30% criterion is that it captures something that reference to median latency alone might miss (Figure 2). Indeed given that the distributions are clearly multimodal, any measure of central tendency must summarise the data poorly. In overlap conditions the two larger groups of participants (CC & WB, both N = 70) appeared to fall into two groups around the 30% ES criterion (Figure 2b,f). Participants with very high proportions of express saccades in overlap conditions (eg >50%) did not occur in the Caucasian group, but did occur in both Chinese groups, confirming our previous observation (see Figure 2 in [19]). In a previous study[16] we were only able to find a single Caucasian ESM making comparison with the Chinese ESMs difficult. However, here we identified seven Caucasian ESMs, although as noted above none of them reached the very high proportions of ES observed in both Chinese groups. However the latency distributions of Chinese and Caucasian ESMs were clearly similar (Figure 4). We would argue that this demonstrates that we are dealing with a single phenomenon (the overproduction of ES in overlap conditions) that occurs at different rates in the two populations we have examined. This implies that we are not simply dealing with a difference between populations, but also a difference within populations.

We have confirmed our previous finding of a difference in a simple, reflexive oculomotor behaviour between Chinese and Caucasian participants. Importantly, while previously participants were tested in different locations (although we made strenuous efforts to standardise methods and procedures), this time all participants were tested in the same location. What then might explain the difference between them? It remains unclear whether, as often claimed, the key variable that might explain such differences is the culture to which participants have been primarily exposed. Many “cultural” neuroscience studies never independently assess the “culture” of participant groups. Participants are recruited from different nationalities [12], [44], or recruited from different locations [15], [45], and cultural differences are assumed. In the present study, rather than assume cultural differences we sought to confirm cultural differences/similarities using the Schwartz Value Survey (SVS).

Schwartz value theory defines ten distinct value types which are claimed to be recognised across different cultures [34], [37], [46]. While the values theory approach to cross-culture comparison has been debated [47]–[49], it offered a number of advantages. The presentation of a large number of items, across different values and attitude types, did not require us to make assumptions about our participant groups. Further, the breadth of the SVS gave us some confidence that differences, if present, would be picked up. The SVS was available, and has previously been used in large studies, in both English and Mandarin, and analysed using standardised procedures [34], [41]. Our objective in using it was not to make specific claims about the content of the culture of our groups, but to establish that there really was a difference between the two Chinese groups, rather than simply assume such a difference.

The value scores as we have presented them show the relative importance/lack of importance participants assign to particular values and allow us to assess the extent to which our different participant groups were similar or dissimilar to each other. The data show that the UK-Chinese participants (Group BC) tended to generate the same pattern of value scores as their UK-Caucasian counterparts (Group WB), and a different pattern to the participant group recruited from among Chinese overseas students studying in the UK (Group CC). Thus, Groups BC and WB tended to report that “power” was less important to them than Group CC, while they reported that “self-direction” was more important relative to Group CC (Figure 1). We make no claim as to how typical Group CC is likely to be of the Chinese population. Indeed clearly they represent a small, highly selected subgroup from within the mainland Chinese population; as a group they have the education, finances, interest and ambition to travel far from home to a different country and culture, and learn in a different language. However, the SVS scores were consistent with our expectation that culturally they are distinct from Group BC. Yet, despite the cultural dissimilarity of the two Chinese groups, their oculomotor performance was identical, and different to that observed in the Caucasian group. Thus, whatever explains the oculomotor differences that we have observed, it is unlikely that it is culture.

While many studies have made binary distinctions between participant groups drawn from Western versus East Asian cultures, a small number have also recruited an intermediate group, usually a group of East Asian parentage, but primarily exposed to Western culture. In a study of global versus local visual attention (East Asian participants are biased towards a global attentional processing style), a group of Asian Australians tended to respond more like East Asians than Caucasian Australians[29]. This is a slightly weaker result than ours in that we found identical performance in the two Chinese groups. However, neither is it consistent with the notion that culture primarily shapes at least this aspect of cognition. Perhaps of more direct relevance, an experiment on eye movement strategies in a face recognition paradigm (East Asians tend to fixate the centre of faces when unconstrained, whereas Caucasians fixate the eye area) demonstrated that a group of British-born Chinese participants persisted with the East Asian strategy [30] (see also [50]). This is consistent with our result, although clearly for a different and more complex aspect of oculomotor control.

There are many factors that might explain the specific oculomotor difference we have observed between different participants groups. However, particularly when considered alongside the reports described above, there is little direct support for the hypothesis that culture plays a key role in shaping low level cognitive and oculomotor behaviour. It is currently unclear what explains the oculomotor difference between Chinese and Caucasians that we have observed in three different experiments [16], [28]. However, if this difference is stable through time (something we have yet to demonstrate), we suggest that what we are describing is a distinct oculomotor phenotype. Given a number of important genetic differences between East Asian and Caucasian populations (in, for example neurotransmitter systems [51], [52]), a fruitful avenue of investigation would be whether the oculomotor differences we have observed maps onto known genetic differences. Potentially, this might provide useful information both about the underlying neurogenetics of the oculomotor system and about human genetic and behavioural diversity.

## Supporting Information

File S1 **Supporting Information.** **Table S1. Schwartz values and their definitional goals.** Goal definitions are taken from [25]. Note these are not the individual items as presented in the Schwartz Value Survey, examples of which are given below. The items are grouped as shown in the third column [27] to generate the value scores. **Figure S1. Examples from the Schwartz Value Survey.** The first five items from the SVS are shown. Participants rated these on a scale from -1 (against my values) to 7 (of supreme importance).
(DOCX)
