# Supplementary material for: Cultural Diversity and Saccade Similarities: Culture Does Not Explain Saccade Latency Differences between Chinese and Caucasian Participants
Source: PLoS One. 2014 Apr 7;9(4):e94424. doi: 10.1371/journal.pone.0094424 (PMC3978040; doi:10.1371/journal.pone.0094424)
Supplement: File S1 — Supporting Information. Table S1. Schwartz values and their definitional goals. Goal definitions are taken from [25]. Note these are not the individual items as presented in the Schwartz Value Survey, examples of which are given below. The items are grouped as shown in the third column [27] to generate the value scores. Figure S1. Examples from the Schwartz Value Survey. The first five items from the SVS are shown. Participants rated these on a scale from -1 (against my values) to 7 (of supreme importance). (DOCX) [file pone.0094424.s001.docx]

| **Value Type** | **Defining Goal** | **Items on SVS** |
| --- | --- | --- |
| Conformity | Restraint of actions, inclinations, and impulses likely to upset or harm others and violate social expectations or norms. | 11, 20, 40, 47 |
| Tradition | Respect, commitment, and acceptance of the customs and ideas that one's culture or religion provides. | 18, 32, 36, 44, 51 |
| Benevolence | Preserving and enhancing the welfare of those with whom one is in frequent personal contact (the ‘in-group’). | 33, 45, 49,52, 54 |
| Universalism | Understanding, appreciation, tolerance, and protection for the welfare of *all* people and for nature. | 1, 17, 24, 26, 29, 30, 35, 38 |
| Self-Direction | Independent thought and action-choosing, creating, exploring. | 5, 16, 31, 41, 53 |
| Stimulation | Excitement, novelty, and challenge in life. | 9, 25, 37 |
| Hedonism | Pleasure or sensuous gratification for oneself. | 4, 50, 57 |
| Achievement | Personal success through demonstrating competence according to social standards. | 34, 39, 43, 55 |
| Power | Social status and prestige, control or dominance over people and resources. | 3, 12, 27, 46, 58 |
| Security | Safety, harmony, and stability of society, of relationships, and of self. | 8, 13, 15, 22, 56 |

**Table S1. Schwartz values and their definitional goals.**

**Figure S2. Examples from the Schwartz Value Survey.**

*1 EQUALITY (equal opportunity for all)*

*2 INNER HARMONY (at peace with myself)*

*3 SOCIAL POWER (control over others, dominance)*

*4 PLEASURE (gratification of desires)*

*5 FREEDOM (freedom of action and thought)*
